# Case Report: Unusual Varicella-Zoster Virus Meningoencephalitis With Meningomyelitis Mimicking Central Nervous System Leukemia

**DOI:** 10.3389/fmed.2022.847219

**Published:** 2022-04-20

**Authors:** Ranran Tu, Jianyang Liu, Fan Cheng, Weipin Weng, Hainan Zhang, Yi Shu, Xiaomei Wu, Zhiping Hu, Jie Zhang

**Affiliations:** Department of Neurology, The Second Xiangya Hospital of Central South University, Changsha, China

**Keywords:** central nervous system infections, Varicella-zoster virus, metagenomic next-generation sequencing, acute myelogenous leukemia, case report

## Abstract

**Background:**

Varicella-Zoster Virus (VZV) is a human pathogen from the α-subfamily of herpesviruses. In immunocompromised patients, VZV may produce disease of the central nervous system (CNS). Clinical manifestations of CNS infection with VZV are non-specific and can mimic other infectious and non-infectious diseases. Due to similar symptoms, CNS infection with VZV represents a diagnostic challenge. Here, we report a case of a patient who showed laboratory and imaging manifestations mimicking the neoplastic etiology.

**Case:**

A 31-year-old man presented with a 3-day history of headache, 5-h of confusion, generalized tonic-clonic seizures, and transient fever. The patient has a history of acute myelogenous leukemia (AML). His cerebrospinal fluid (CSF) studies revealed markedly elevated protein (10.326 g/L) and atypical cells. Meanwhile, the MRI of brain, cervical, and thoracic spine was reported as extensive (frontal, parieto-occipital and temporal pachymeningeal, and falx cerebri) enhancement and irregular thickening. These examinations suggested a suspicion of CNS involvement of AML. However, based on further investigations with metagenomic next-generation sequencing, a final diagnosis of VZV meningoencephalitis with meningomyelitis was made. With acyclovir and foscarnet sodium therapy, repeated CSF studies revealed normal cell count and protein. No atypical cells were found. The repeated brain MRI also revealed obvious resolution of the previous abnormal pachymeningeal enhancement.

**Conclusion:**

This case highlights the importance of recognizing the unusual phenomenon of traditional tests in VZV meningoencephalitis with meningomyelitis, and timely using of further precise examinations to detect viral DNA, which is required to prevent missed diagnosis.

## Introduction

Varicella-zoster virus (VZV), also known as human herpesvirus 3, is a member of the herpesviridae family of double-stranded DNA viruses, which is associated with an age-mediated decrease in cell-mediated immunity and under immunosuppression. The primary infection causes varicella, after which this virus becomes latent in the sensory ganglia of the cranial nerve or the dorsal root ganglia, and enteric and autonomic ganglia. VZV can travel retrograde to produce meningoencephalitis, myelitis, and stroke (1). The neurological complications of VZV infections are more common in immunosuppressed individuals. VZV meningitis often presented with mild cerebrospinal fluid (CSF) pleocytosis and a slight increase in protein levels. Only rare cases are associated with isolation of the causative virus from the CSF. Detection of VZV DNA in CSF has confirmed VZV as a cause of meningitis or meningoencephalitis (2).

We present an unusual case of an adult male with a history of acute myelogenous leukemia (AML) who presented to our hospital with headaches, confusion, and seizure. After a comprehensive workup, he was diagnosed with VZV meningoencephalitis with meningomyelitis and achieved significant improvements after the combinations of acyclovir and foscarnet sodium therapy.

## Case Description

**Figure 1:**
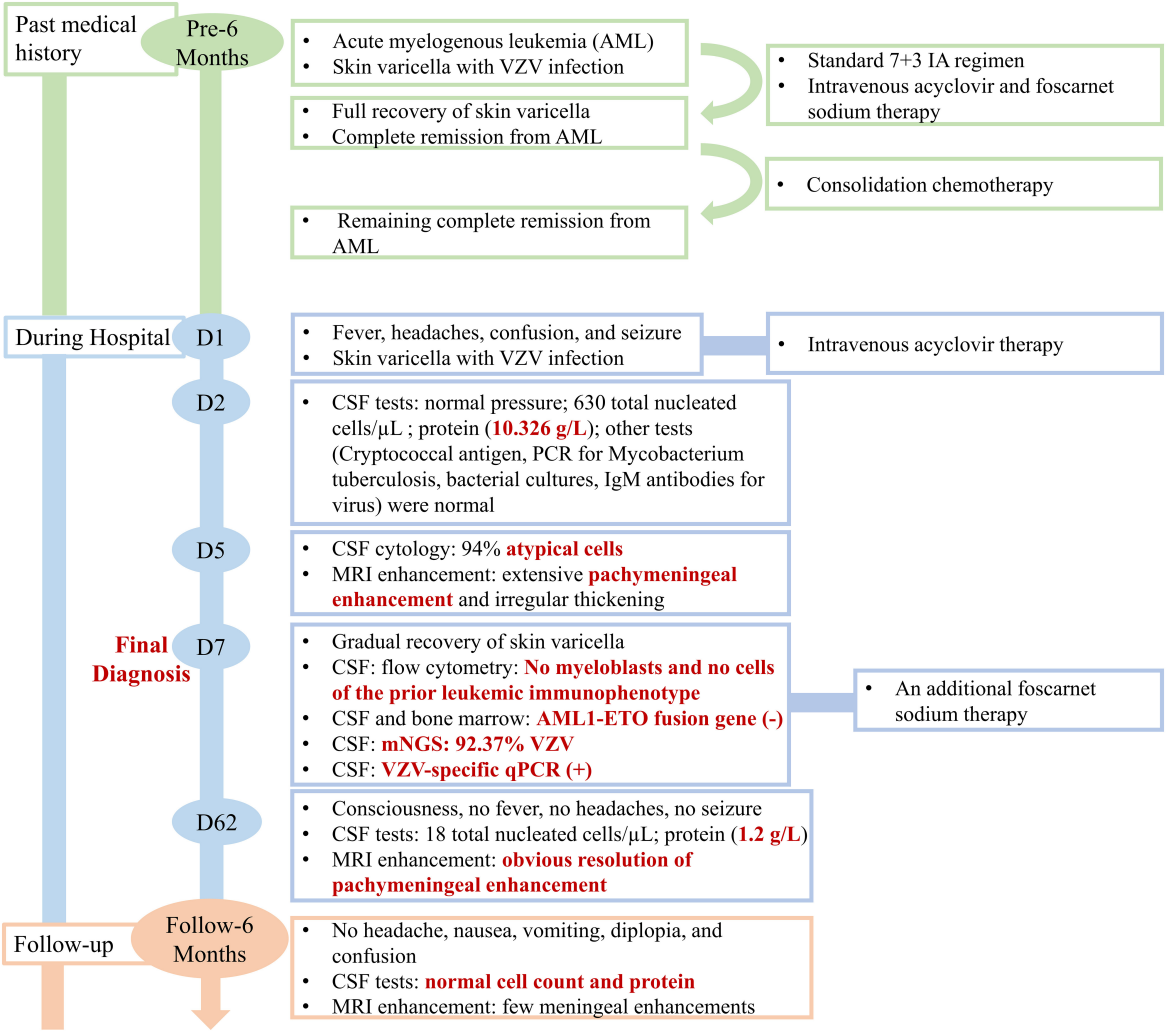
The clinical course of the patient (schematic).

A 31-year-old man presented to the emergency department with a 3-day history of headache along with nausea and vomiting, 5 h of confusion followed by generalized tonic-clonic seizures and fevers to 38.9°C. His seizures occurred twice within an hour, lasting about 5 min and 2 min each and resolving spontaneously. The patient did not have personal or family history of seizures. His past medical history was significant for an AML with t (8; 21) (q22; q22.1)/RUNX1-RUNX1T1 6 months prior. Meanwhile, when he was first diagnosed with AML, he also had a widespread papular rash with vesicles in the lip, trunk, and extremities, which suggested varicella with VZV infection. With the diagnosis of AML, the patient received a standard 7 + 3 IA regimen (idarubicin 10 mg/m^2^ for 3 days, cytarabine 100 mg/m^2^ for 7 days) as induction chemotherapy which achieved complete remission. Considering the VZV infection, the patient simultaneously received intravenous acyclovir and foscarnet sodium, after which the skin lesions were fully recovered. During regular chemotherapy, although the CSF pressure and routine analysis were normal, the intrathecal chemotherapy with cytarabine, methotrexate, and dexamethasone was prophylactically given. Consolidation chemotherapy with another cycle of IA and 2 cycles of high dose cytarabine was administered, and he remained in complete remission. An outline of the episodes is described in Figure 1.

On admission, his vital signs included a temperature of 37.5°C. A skin examination revealed a few scattered erythematous rash and varicella in the lip and extremities. In the neurologic examination, the patient was confused and unable to follow complicated commands. Withdrawal reflex response was elicited by noxious stimuli to the upper and lower extremities. Pupils were reactive, but he displayed impaired left eye adduction and right eye abduction. He had signs of meningeal irritation in the form of neck stiffness, positive Kernig's, and Lesage's sign. Movement and sensory examination were unreliable due to the patient's altered level of consciousness. He had a palpable bladder. The Babinski sign was positive bilaterally. CT scans of the head showed no acute abnormalities. Considering the skin varicella, intravenous acyclovir (at a dose of 10 mg/kg q8 h) therapy was instituted on day 1 after admission. However, it is doubtful whether the skin varicella on admission was the reactivation of VZV.

Based on the initial presentation, a broad differential diagnosis was considered. First, we should consider the localization for his presentation. The symptoms of headache, confusion, and seizures clinically suggested a central rather than peripheral etiology. Neck stiffness indicates involvement of the meninges, and generalized tonic-clonic seizures suggest diffuse cortical involvement. The urinary retention suggests either bi-hemispheric lesions or a spinal cord lesion; Second, we should consider the etiology of his presentation. The differential etiology included infection (e.g., meningoencephalitis, meningomyelitis, and progressive multifocal leukoencephalopathy), intracranial malignancy (e.g., CNS leukemia, leptomeningeal carcinomatosis, and paraneoplastic syndromes), toxic encephalopathy (e.g., chemotherapy-induced), inflammatory etiologies (e.g., autoimmune encephalitis and acute disseminated encephalomyelitis), and vascular (e.g., cerebral venous sinus thrombosis).

On day 2 after admission, the lumbar puncture revealed xanthochromic CSF with a slow flow rate. The patient's CSF opening pressure was normal (140 mm H_2_O). CSF analysis showed pleocytosis (630 total nucleated cells/μl with 70% mononuclear cells), marked elevation in protein at 10.326 g/L, normal glucose/chloride, negative Gram stain, negative Cryptococcal antigen, negative PCR for Mycobacterium tuberculosis, and negative bacterial cultures. Immunoglobulin M (IgM) antibodies for herpes simplex virus, Epstein–Barr virus, and cytomegalovirus were negative.

**Figure 2:**
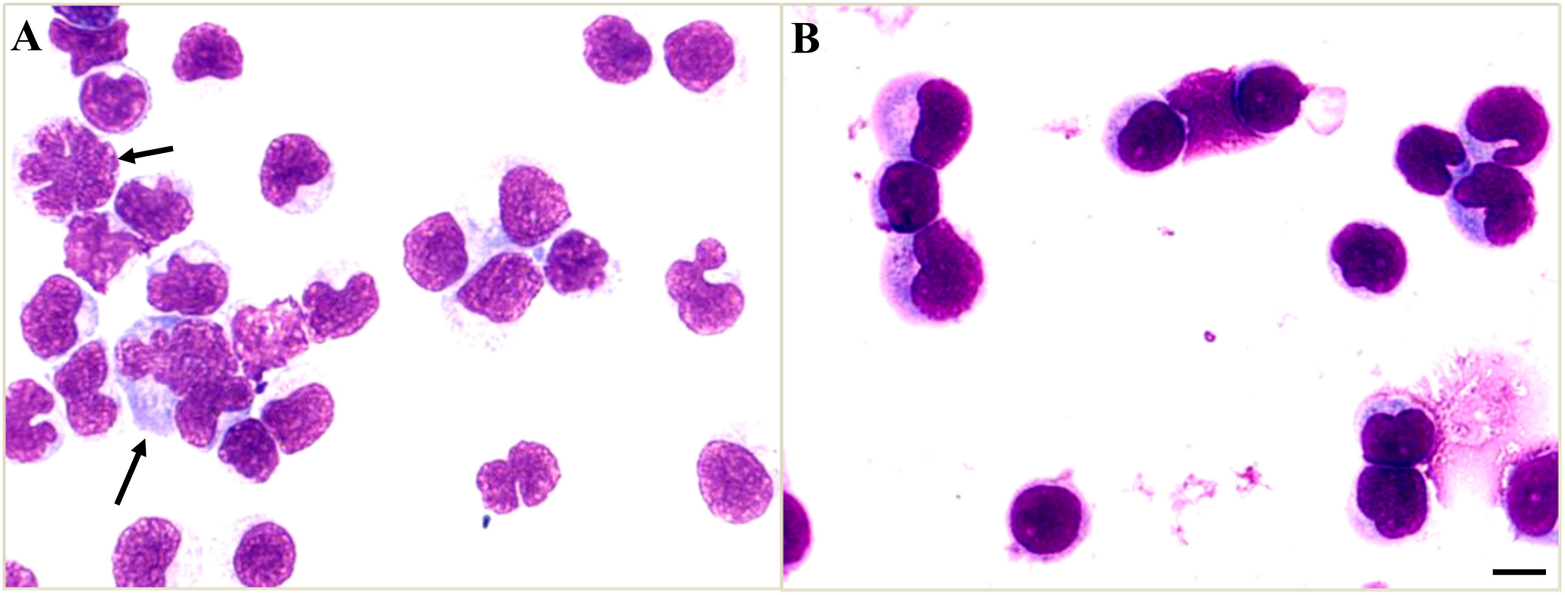
Cerebrospinal fluid (CSF) cytology shows atypical cells. **(A)** The atypical cells of CSF on day 5 after admission were scattered and varied in size, the cell membrane was malformed, and had a high nuclear-cytoplasmic ratio (black arrows). **(B)** The CSF cytology on day 19 after admission showed reactive lymphocytes and monocytes. Scale bars: 100 μm.

**Figure 3:**
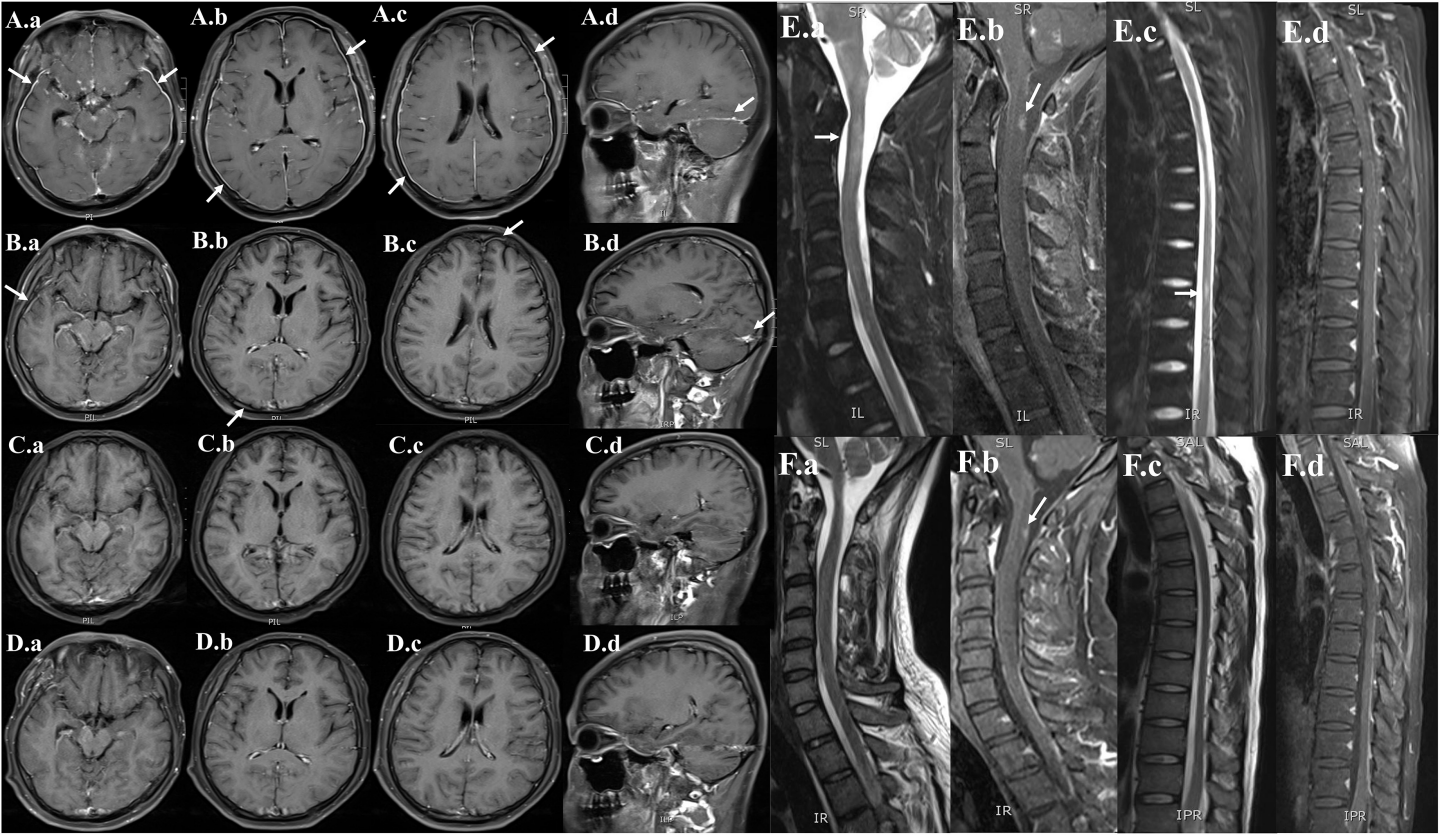
Serial neuroimaging with MRI demonstrating lesion amelioration. **(A)** Contrast-enhanced MRI brain (hospital day 5), (a,b,c) axial and (d) sagittal T1-weighted images showing diffuse leptomeningeal and pachymeningeal contrast enhancement (white arrows). **(B)** Repeat MRI on hospital day 29, (a,b,c) axial and (d) sagittal views showing less meningeal enhancement after 3 weeks of therapy. **(C)** Repeat MRI on the time of discharge (hospital day 62), (a,b,c) axial and (d) sagittal views showing the obvious resolution of the previous meningeal enhancement. **(D)** Repeat MRI on 6-month follow-up (a,b,c) axial and (d) sagittal views showing no meningeal enhancements. **(E)** MRI of the cervical and thoracic spine (hospital day 5), (a,c) sagittal T2-weighted and (b,d) post-contrast T1-weighted showing abnormal longitudinally extensive T2 weighted hyperintensities involving the C2–C3 and T6–T9. Partial enhancement was observed in the dorsal aspect of the spinal cord at C2 and T6–T7 levels (white arrows). **(F)** Repeat MRI on hospital day 29, (a,c) sagittal T2-weighted and (b,d) post-contrast T1-weighted images showing lesser abnormal hyperintensities compared with previously.

On day 5 after admission, the cytology testing of CSF revealed 4% lymphocytes, 2% monocytes, and 94% atypical cells (Figure 2A). Brain MRI with contrast was reported as multiple abnormal signals in the cortex, extensive pachymeningeal enhancement, and irregular thickening (Figure 3A). The Magnetic resonance venography and diffusion-weighted imaging were normal. MRI of the cervical and thoracic spine showed T2 hyperintense lesions located at C2–C3 and T6–T9, with partial enhancement (Figure 3E).

Based on the above examinations, we have a debate about the diagnosis. The fever and apparent immunocompromise support infectious causes. CSF profile of pleocytosis, high protein level, and normal glucose/chloride helped to confine the differential diagnosis to virus, bacteria, tuberculosis, and fungi. The serum and CSF screens for bacteria, tuberculosis, and fungi were normal. Thus, we excluded the CNS infections with bacteria, tuberculosis, and fungi. The varicella infection supported the suspicion of meningoencephalitis associated with VZV. However, brain MRI of our patient showed obvious enhancement and thickening of pachymeninges. The pachymeningeal enhancement was different from the typical MRI imaging of viral meningitis, which often presented with diffuse enhancement along the leptomeninges. Furthermore, his CSF analysis showed marked elevation in protein at 10.326 g/L, which is rare in viral infections. Moreover, CSF cytology suggested 94% atypical cells. Based on his past medical history, the suspected presence of atypical cells in CSF raised the concern for CNS leukemia. The above diagnostic workup was unable to differentiate between VZV infections and CNS leukemia. Thus, additional testing should be performed.

The flow cytometric analysis was proved to be a sensitive test for detecting CNS involvement when compared to traditional CSF cytology (3). On day 7 after admission, cell phenotypes of immune cells were detected by flow cytometry from CSF. The cell-surface markers used in flow cytometry include CD45, CD14, CD64, CD117, CD3, CD4, and CD19, which showed a predominance of mature lymphocytes (93.6%) and rare monocytes (0.7%). No myeloblasts and no cells of the prior leukemic immunophenotype were identified. Thus, we speculated that the atypical cells in CSF cytology do not belong to the malignant clone but are reactive. Furthermore, the *AML1–ETO* fusion gene in CSF and bone marrow was also negative. The negative results of flow cytometry and *AML1–ETO* fusion gene from CSF should cast doubt on the diagnosis of CNS leukemia.

**Figure 4:**
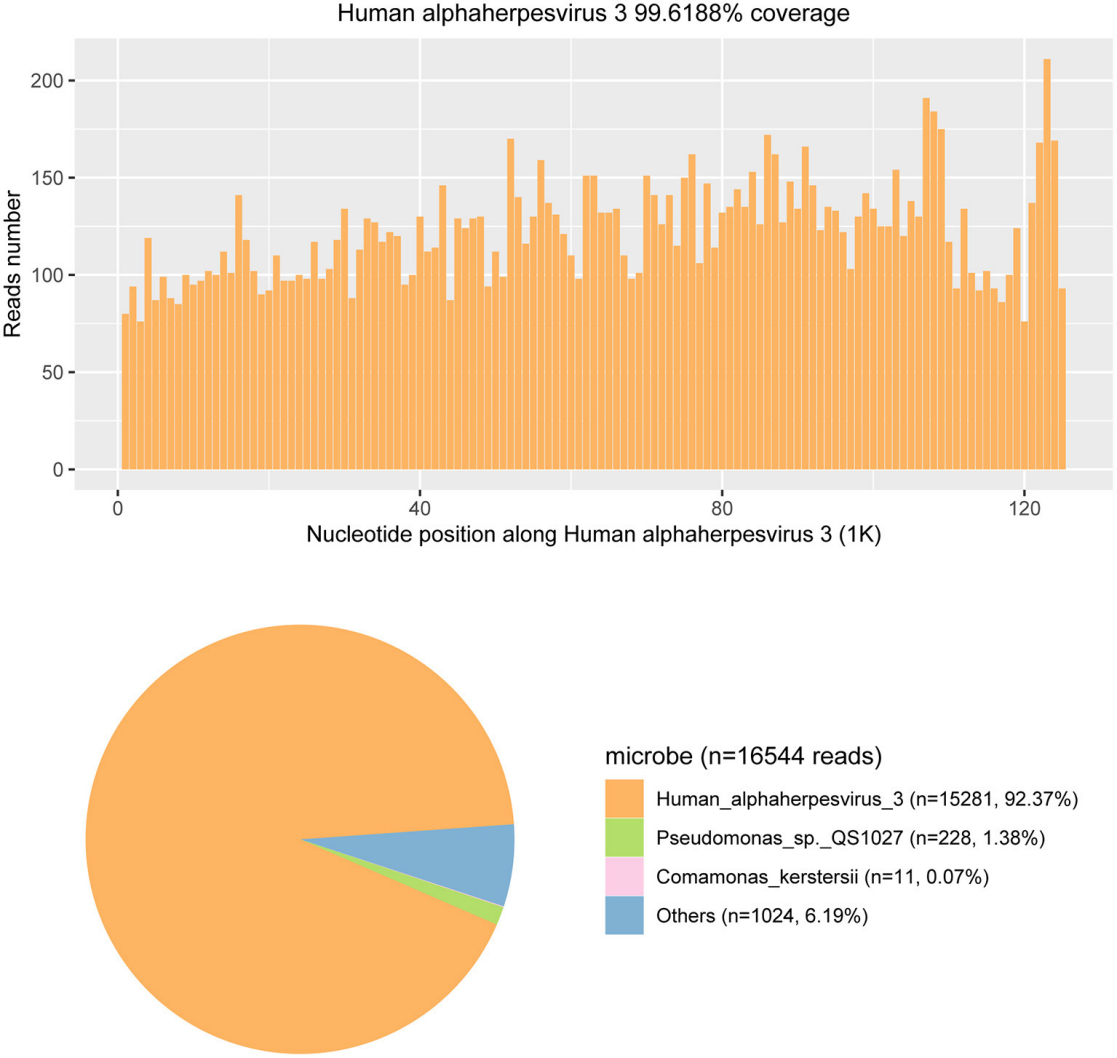
The metagenomic next-generation sequencing (mNGS) results of pathogen identification. Approximately 92.37% of viral reads corresponded to Human alphaherpesvirus 3 (also named the Varicella-zoster virus) with coverage of 99.6188% in CSF.

Given the strong suspicion for viral meningoencephalitis, other CSF tests were also conducted on day 7 after admission. On this set of CSF studies, metagenomic next-generation sequencing (mNGS) and quantitative real-time polymerase chain reaction (qRT-PCR) were performed to further assess for an infectious cause. The mNGS has the capability of rapid, sensitive, and accurate pathogen identification (4). The sequencing detection identified 15,281 (out of 16,544) sequence reads uniquely aligned to the VZV genome, and these reads covered a high percentage (92.37%) of the VZV (Figure 4). The presence of VZV DNA in CSF was further verified by VZV-specific qRT-PCR. The CSF qRT-PCR was positive for VZV.

Based on the positive mNGS and confirmatory qRT-PCR test, a final diagnosis of VZV meningoencephalitis with meningomyelitis was made. The patient received an additional foscarnet sodium (3g q12 h) on day 7 after admission. The combined use of acyclovir and foscarnet sodium resulted in a steady improvement in symptoms. After combination therapy, repeated CSF studies on day 19 after admission revealed 95% lymphocytes and 5% monocytes. Meanwhile, no atypical cells were found in the cytology testing of CSF (Figure 2B). On day 7 after admission, the skin varicella showed gradual recovery. A repeated MRI on day 29 after admission showed less meningeal enhancement (Figures 3B,F). During the entire treatment course of hospitalization, we did not use steroids.

By the time of discharge (hospital day 62), the patient was conscious, and denied headache fever. No fever and seizure occur during hospitalization. Examination revealed normal eye movements, normal strength/sensation, normal coordination/gait testing, and no nuchal rigidity. But the Kernig's sign and left-sided Babinski's sign were still positive. Repeated CSF studies revealed a clear appearance, pleocytosis (18 total nucleated cells/μl), and elevated protein (1.2 g/L). Compared with the CSF on admission, the pleocytosis and elevated protein were significantly alleviated. A repeated brain MRI revealed obvious resolution of the previous abnormal pachymeningeal enhancement (Figure 3C). At the 6-month follow-up visit, he denied headache, nausea, vomiting, diplopia, and confusion. Repeated CSF studies revealed normal cell count and protein (Table 1). The repeated brain MRI revealed no abnormal meningeal enhancement (Figure 3D).

**Table 1:** Cerebrospinal fluid (CSF) profile at 4 different time points during hospitalization and 6-month follow-up.

	**Cell count (10^**∧**^6/L)**	**Protein (mg/L)**	**Glucose (mmol/L)**
**Before acyclovir and foscarnet sodium therapy**			
Hospital day 2	630 (70% mononuclear cells)	10,326	4.18
Hospital day 5	580 (95% mononuclear cells)	6,966	4.54
**After acyclovir and foscarnet sodium therapy**			
Hospital day 19	56 (80% mononuclear cells)	4,049	3.58
Hospital day 62	18 (12/18 mononuclear cells)	1,244	3.28
**Follow-Up**			
6-Month	0	418	3.53

## Discussion

Our patient's VZV infections and history of AML are interesting confounders. Neurologic signs and symptoms may develop in AML from direct involvement of CNS, which includes parenchymal and leptomeningeal dissemination, myeloid sarcoma, and neuroleukemiosis. Meanwhile, immunosuppression secondary to AML leads to an increased risk of VZV infections. The signs and symptoms of viral meningoencephalitis with meningomyelitis may mimic CNS leukemia. The manifestations of CNS leukemia are diverse, such as headaches, cranial nerve dysfunction, altered mental status, and radicular pain (5). In our patient, the clinical manifestations with fever, confusion, headaches, seizure, and positive signs of meningeal irritation are non-specific and can present in both viral meningoencephalitis with meningomyelitis and CNS leukemia. Due to the similar manifestations, differentiating between CNS leukemia and viral infections is often difficult.

Although VZV disease caused by VZV reactivation is a common infectious complication after leukemia, meningoencephalitis with meningomyelitis is rare. In the literature, only a few cases reported the VZV meningoencephalitis in patients with a history of AML (6–8) or chronic myeloid leukemia (9). One case reported a patient who underwent allogeneic hematopoietic transplantation for AML, whose CSF cytology showed atypical reactive monocytic cells (8). The protein level in these cases showed markedly elevated at 323.3 mg/dl (6), 1,064 mg/dl (7), or 7.3 g/L (9).

In our patient, the CSF cytology also showed atypical cells, which were identified as reactive cells instead of malignant cells by subsequent flow cytometry. The CSF atypical cells in viral infections are relatively uncommon, which reported in Epstein–Barr virus (10), enteroviruses (11), West Nile Virus (12), and VZV (8). This phenomenon was referred to as a cerebrospinal leukemoid reaction (8, 13, 14), which suggested the atypical cells in CSF are reactive virocytes (cells with different morphologies) and may result from severe infections. With the acyclovir and foscarnet sodium therapy, the CSF pleocytosis and atypical cells may be gradually resolved.

Furthermore, the CSF protein level in our case is also significantly increased (10.326 g/L), which gradually decreased after acyclovir therapy. Generally, high protein concentration in the CSF is non-specific. However, a protein level of more than 10 g/L in viral meningoencephalitis is rare, which is mainly reported in HIV-positive patients (15, 16) and patients with leukemia (7) or malignant lymphoma (17). Particularly, in a patient with a history of AML, the markedly elevated protein in CSF may suggest CNS leukemia rather than viral etiology. Our case emphasizes the utility of taking mNGS and qRT-PCR to detect potential viral causes, even the extremely elevated protein level in CSF. Moreover, brain MRI in our patient showed diffuse pachymeningeal enhancement, which is an unusual meningeal enhancement in viral meningoencephalitis. Repeat brain MRI after acyclovir therapy revealed obvious resolution of the abnormal pachymeningeal enhancement. In total, there are three points in our case that differ from most viral meningoencephalitis with meningomyelitis: (1) the presence of CSF atypical cells. (2) The extremely high protein concentration in the CSF (more than 10 g/L). (3) The diffuse pachymeningeal enhancement in brain MRI.

With the diagnosis of VZV meningoencephalitis with meningomyelitis, the patient received the combination therapy acyclovir and foscarnet sodium. In the literature, the American Society of Clinical Oncology (ASCO) and the Infectious Disease Society of America (IDSA) recommend that patients who are seropositive for herpes simplex virus with leukemia receive antiviral prophylaxis (18). Thus, for the patients with seropositive/seronegative for HSV, the prophylactic strategy should be given at the time of induction therapy.

## Conclusion

We reported an uncommon case of VZV meningoencephalitis with meningomyelitis whose CSF and MRI findings initially concerning for CNS involvement of AML. Moreover, we underlined that the phenomenon of extremely elevated protein and atypical cells in CSF and pachymeningeal enhancement in brain MRI, even in patients with a history of malignant disease, warrants further precise tests, and consultation with the clinician.

## Data Availability Statement

The original contributions presented in the study are included in the article/Supplementary Material, further inquiries can be directed to the corresponding author/s.

## Ethics Statement

Written informed consent was obtained from the individual(s) for the publication of any potentially identifiable images or data included in this article.

## Author Contributions

JZ conceived the idea of this article. RT and JL contributed to the preparation of the manuscript and interpreting the data of the patients. All authors contributed to review, edit, read, and approved the final manuscript.

## Conflict of Interest

The authors declare that the research was conducted in the absence of any commercial or financial relationships that could be construed as a potential conflict of interest.

## Publisher's Note

All claims expressed in this article are solely those of the authors and do not necessarily represent those of their affiliated organizations, or those of the publisher, the editors and the reviewers. Any product that may be evaluated in this article, or claim that may be made by its manufacturer, is not guaranteed or endorsed by the publisher.
